# Genetic Analysis of TB Susceptibility Variants in Ghana Reveals Candidate Protective Loci in *SORBS2* and *SCL11A1* Genes

**DOI:** 10.3389/fgene.2021.729737

**Published:** 2022-02-15

**Authors:** Adwoa Asante-Poku, Portia Morgan, Stephen Osei-Wusu, Samuel Yaw Aboagye, Prince Asare, Isaac Darko Otchere, Samuel Mawuli Adadey, Khuthala Mnika, Kevin Esoh, Kenneth Hayibor Mawuta, Nelly Arthur, Audrey Forson, Gaston Kuzamunu Mazandu, Ambroise Wonkam, Dorothy Yeboah-Manu

**Affiliations:** ^1^ Noguchi Memorial Institute for Medical Research, University of Ghana, Accra, Ghana; ^2^ West African Centre for Cell Biology of Infectious Pathogens, University of Ghana, Accra, Ghana; ^3^ Department of Biochemistry, Cell, and Molecular Biology, University of Ghana, Accra, Ghana; ^4^ Division of Human Genetics, Faculty of Health Sciences, University of Cape Town, Cape Town, South Africa; ^5^ Department of Chest Diseases, Korle-Bu Teaching Hospital Korle-Bu, Accra, Ghana

**Keywords:** tuberculosis, SLC11A1, Sorbs2, Ghana, genomics

## Abstract

Despite advancements made toward diagnostics, *tuberculosis* caused by *Mycobacterium africanum* (Maf) and *Mycobacterium tuberculosis sensu stricto* (Mtbss) remains a major public health issue. Human host factors are key players in *tuberculosis* (TB) outcomes and treatment. Research is required to probe the interplay between host and bacterial genomes. Here, we explored the association between selected human/host genomic variants and TB disease in Ghana. Paired host genotype datum and infecting bacterial isolate information were analyzed for associations using a multinomial logistic regression. *Mycobacterium tuberculosis* complex (MTBC) isolates were obtained from 191 TB patients and genotyped into different phylogenetic lineages by standard methods. Two hundred and thirty-five (235) nondisease participants were used as healthy controls. A selection of 29 SNPs from TB disease-associated genes with high frequency among African populations was assayed using a TaqMan® SNP Genotyping Assay and iPLEX Gold Sequenom Mass Genotyping Array. Using 26 high-quality SNPs across 326 case-control samples in an association analysis, we found a protective variant, rs955263, in the *SORBS2* gene against both Maf and Mtb infections (*P*_*BH*_ = 0.05; OR = 0.33; 95% CI = 0.32–0.34). A relatively uncommon variant, rs17235409 in the *SLC11A1* gene was observed with an even stronger protective effect against Mtb infection (MAF = 0.06; PBH = 0.04; OR = 0.05; 95% CI = 0.04–0.05). These findings suggest SLC11A1 and SORBS2 as a potential protective gene of substantial interest for TB, which is an important pathogen in West Africa, and highlight the need for in-depth host-pathogen studies in West Africa.

## Introduction

Pulmonary *tuberculosis* (TB), a disease of public health importance, is caused by pathogenic members of the *Mycobacterium tuberculosis* complex (MTBC), such as *M. africanum* (Maf) and *M. tuberculosis sensu stricto* (Mtbss). Each species comprising several clades of specific strains show variable virulence and disease-causing mechanisms (Yeboah-Manu et al., 2011; Delogu et al., 2013; Reiling et al., 2013; Brites and Gagneux, 2015). While Maf is restricted to West African countries including Ghana, Mtb is responsible for TB cases worldwide (Gagneux and Small, 2007). As a disease transmitted through aerosol, (Delogu et al., 2013), it is expected that susceptible immunocompetent individuals who come into contact with aerosol(s) containing viable bacteria will be equally infected. However, in about 90% of infected individuals, the host immune system can wall off the site of infection in a granuloma (Ghon complex) (Malik and Godfrey-Faussett, 2005; Gagneux et al., 2008; Yeboah-Manu et al., 2011). However, with the remaining 10%, only 5% will potentially develop active TB disease within 2–3 years of infection, while the remaining 5% may develop TB later in their life (Sakamoto, 2012; Fattorini et al., 2013; Narasimhan et al., 2013). This unique differential infection propensity depends on the interplay between the genetic makeup of the human host (Thye et al., 2010; Oki et al., 2011; Thye et al., 2012; Chimusa et al., 2014; Kinnear et al., 2017), the environment, and socioeconomic factors (Cheng et al., 2017; Mohidem et al., 2018).

In recent years, genetic association studies between important genes and susceptibility to infectious diseases such as TB, Malaria, and HIV using linkage and genome-wide association (GWAS) have been reported (Comstock, 1978; Pydi et al., 2013; McHenry et al., 2020). With these studies, several genes have been reported to be associated with TB, including Toll-like receptors (TLRs), cytokines/chemokines, and their receptors leukocyte antigens (HLAs), SLC11A1, major histocompatibility complex-(MHC), mannose-binding lectin (MBL), vitamin D Receptor (VDR), etc. (Søborg et al., 2003; Salie et al., 2013). For instance, in 2012, Pydi et al. reported on inhibitory genes, such as KIR3DL1 and KIR2DL3, that confer susceptibility toward TB in individuals.

Although these studies provide useful insights into the disease etiology, very few of these studies investigated the association between these gene variants and specific MTBC strains (McHenry et al., 2020). For example, in a cohort study of 1,916 sputum-positive Ghanaian TB patients genotyped for the ALOX5 g.760G > A variant, individuals with heterozygous alleles were found to be at increased risk for developing TB (Herb et al., 2008). Through stratification by MTBC lineage, this association was mainly driven by patients harboring the exonic variant (g.760A) and infected with Maf [OR = 1.70 (95% CI: 1.2–2.6)] (Herb et al., 2008). Conversely, a protective association [OR = 0.60 (95% CI: 0.4–0.9)] was identified among the occurrence of TB caused by Maf but not Mtb and the mannose-binding lectin (MBL2) G57E variant in another cohort of Ghanaian patients (Thye et al., 2011). Moreover, this latter study also found that Maf binds human recombinant MBL more efficiently, perhaps leading to an improved uptake of Maf by macrophages and selection of deficient MBL variants among human populations exposed to Maf.

Comparative analysis of HLA alleles and the Mtb strains isolated from pulmonary TB patients in a South African study found a significant association of HLA-B27 allele with decreased risk of TB caused by the Beijing strain (Salie et al., 2013). Using a candidate gene approach, from a cohort of 237 adult Vietnamese TB patients, Caws et al. concluded that individuals carrying the C allele of the Toll-like receptor-2 (TLR2) T597C polymorphism were significantly more likely to develop TB caused by mycobacteria belonging to the East-Asian/Beijing strain family [OR = 1.57 (95% CI 1.15–2.15)] (Caws et al., 2008). Similar studies have been conducted to understand the coevolution of MTBC and its human host, with regard to the association between different MTBC strains and disease severity. Findings from one such study in Uganda reported an interaction between a single-nucleotide polymorphism (SNP) in SLC11A1 and the lineage (L) L4 of Mtb with several IL12B polymorphisms associated with disease severity (McHenry et al., 2020).

Even though the Sub-Saharan African continent is home to the highest diversity of humans as well as the MTBC, studies to explore the potential association of specific host gene variants and susceptibility to strains of the MTBC are limited (Mboowa, 2014; Omae et al., 2017; McHenry et al., 2020). Thus, host genetics and susceptibility to distinct MTBC lineages cannot be overlooked. Findings from two independent molecular epidemiological studies by our group and other groups (Asante-Poku et al., 2015; Asante-Poku et al., 2016) showed a strong association between Maf (driven by lineage 5) and a native West African ethnic group. To improve our understanding of the genetic susceptibility to the MTBC clades, this current study thence explored genotyping data from the host and pathogen to screen for clade-specific genetic associations in cohorts originating from Ghana.

## Materials and Methods

### Ethical Approval

The study was performed in accordance with the Declaration of Helsinki. Ethical approval for the study was obtained from the Noguchi Memorial Institute for Medical Research Institutional Review Board (NMIMR-IRB 097/15–16). Written and signed informed consent was obtained from participants before enrolment into the study.

### Study Population

From July 1, 2016 to July 31, 2018, this case-control study enrolled participants. The study population was in two categories: One group was made up of only newly diagnosed TB patients (cases), and the other group was made up of healthy individuals serving as the control group (NTB). For the case group, only newly diagnosed sputum smear-positive adult TB cases, registered at the Department of Chest Disease, Korle-Bu Teaching Hospital in Ghana, were recruited into the study before the commencement of TB treatment. All patients were unrelated. Sample size for the study was computed using the EPITOOLS webtool (https://epitools.ausvet.com.au/casecontrolss) for case-control studies based on odds ratios. The parameters furnished to the algorithm included the expected proportion exposed in controls (5%), assumed (minimum) odds ratio, confidence interval (95%), and desired power (80%). Based on these values, a sample size of 348 was estimated, 174 for each group. Therefore, 191 cases and 235 controls were recruited in anticipation of quality filters.

For the control group, five sites of the community by the national TB control program (NTP) were selected. These sites included Korle-gonno (Ablekuma south sub metro), Bukom (Ashiedu Keteke sub metro), Abossey-okai (Okaikoi south), Glefe (Ablekuma West), and Amanfrom (Ga West District). Medical outreaches were conducted in these sites and with a new structured questionnaire, clinical characteristics [diabetes (American Diabetes Association, 2016), HIV, hypertension], and demography and epidemiological data were obtained from each participant using a structure questionnaire. Only patients with no history of TB were recruited into the study. Participants with elevated stage 1 or 2 hypertension were transported to the nearest hospital for immediate medical attention and excluded from the study.

### Sample Collection

#### Diagnosed Tuberculosis Patients Group

To confirm the initial diagnosis at the health facility and to identify the infecting mycobacterial species, a sputum specimen was collected from each TB study participant, following the NTP guidelines. Samples were taken only after participants’ written or thumb-printed consent. Clinical characteristics (previous history of TB, diabetes, BCG vaccination, HIV) as well demography and epidemiological data including age, sex, substance abuse, education, education and ethnic origin were obtained from each participant using a structured questionnaire.

#### Non-tuberculosis Patients Control Group and Chest X-Ray Screening

The screening of eligible control individuals was done in a stepwise manner. First, adults (>18 years) presenting at any of the outreach centers were screened for diabetes and hypertension. Individuals with no evidence of high body temperature (38°C or above) diabetes or elevated hypertension were then taken through chest x-ray (CXR) screening using CAD4TB (version 3.07, Diagnostic Image Analysis Group, The Netherlands) for abnormalities suggestive of pulmonary TB. The software has two abnormality detection systems (textural abnormality and shape abnormality systems), which analyze abnormalities in the unobscured lung fields that have been segmented automatically (Zaidi et al., 2018). A higher score is suggestive of TB. A CAD4TB threshold score of 60 was used for this population determined using previously collected CXR data in a similar population. Sputum samples were collected from all individuals with high CAD4TB scores (60 or greater) and transported to the laboratory for further analysis. Individuals with high CAD4TB scores were referred to the hospitals for further clinical evaluation. Whole blood (5 ml) was collected from each TB patient for host genetics analysis.

### Laboratory Analysis

#### Isolation and Characterization of *Mycobacterium* spp.

Sputum samples obtained were decontaminated using 5% oxalic acid (Yeboah-Manu et al., 2001) and inoculated on two pairs of Lowenstein–Jensen (LJ) slants: one supplemented with 0.4% sodium pyruvate to enhance the isolation of Maf and *M. bovis*, and the other with glycerol for the growth of Mtb. The cultures were incubated at 37°C and were observed weekly for growth for a maximal duration of 16 weeks. MTBC strains were identified by PCR detection of insertion sequence IS*6110* as previously described (Yeboah-Manu et al., 2004). Colonies from positive cultures were purified and stored at −80°C in 2 ml of Middlebrook 7H9 supplemented with ADC enrichment media until use. Pure bacteria DNA was extracted for genotyping using a modified protocol (Otchere et al., 2016) and stored at −20°C until further use.

All MTBC isolates were further typed by spoligotyping (Kamerbeek et al., 1997). This was performed according to the instructions of the manufacturer, using commercially available kits (Isogen Bioscience BV Maarssen, The Netherlands). Briefly, The DR-containing region was amplified by PCR using primers DRa and DRb (GGT​TTT​GGG​TCT​GAC​GAC and CCGAGGGGACGGAAAC). The amplified products were hybridized to a set of 43 oligonucleotides each corresponding to one spacer, immobilized on a nylon membrane. Detection of hybridization was achieved using chemiluminescent ECL (Amersham) liquid followed by x-ray exposure. The spoligotyping patterns obtained were defined according to the SITVITWEB database (http://www.pasteur-guadeloupe.fr:8081/SITVIT_ONLINE). SITVITWEB-assigned shared type numbers were used whenever a spoligotyping pattern was found in the database, while families and subfamilies were assigned based on the MIRU-VNTR*plus* database (http://www.miru-vntrplus) (http://www. miru-vntrplus.org). Shared types were defined as patterns common to at least two or more isolates. All patterns that could not be assigned were considered orphan spoligotypes.

#### Host DNA Isolation

DNA was extracted within 24 h from peripheral blood, following instructions on the available commercial kit Gentra Puregene Blood Kit (QIAGEN) in accordance with the recommendations of the manufacturer. All DNA samples were stored at 80**°**C prior to genotyping. DNA quality was evaluated according to the 260/280 ratio with a Nanodrop 2000 spectrophotometer (Thermo Scientific). In total, 792 samples with concentrations of 5–851.87 µg/µl were selected and sent for single-nucleotide polymorphism (SNP) typing.

#### Host Genetic Analysis: Genotyping of Targeted Single-Nucleotide Polymorphisms in Tuberculosis Disease-Related Host Genes

Targeted genotyping was conducted in the Division of Human Genetics Laboratory, Faculty of Health Sciences, at the University of Cape Town, South Africa. Genetic variants that are potentially associated with MTB were identified from the recent literature. Once the SNPs of interests were identified, we investigated their allele frequencies in African populations present in the 1000 Genomes project (http://www.internationalgenome.org/home) and further narrowed the selection to SNPs that showed high frequency among African populations. This resulted in the selection of 29 SNPs from MTBC-related genes that were investigated in the present study (Supplementary Table S1)*.* SNPs were genotyped using a TaqMan® SNP Genotyping Assay and TaqMan® Universal Master Mix (Life Technologies, Carlsbad, CA, USA), at the Division of Human Genetics, Faculty of Health Sciences, University of Cape Town and by iPLEX Gold Sequenom Mass Genotyping Array (Inqaba Biotec, Pretoria, South Africa). Validation was done in a subset of samples (10%) by Sanger sequencing using BigDye terminator mix (Promega, Madison, WI, USA).

#### Quality Assessment

The genotype dataset was captured in Excel and transformed to PLINK ped + map format using a custom script. SNP coordinates were obtained by querying the ENSEMBL GRCh38 web browser. PLINK binary format files (bed + bim + fam) were then generated using PLINK2, while aligning against the human reference genome in GRCh38 coordinate. Quality checks involved removal of samples for which one or both parents were non-Ghanaians, SNPs with genotyping call rate <90%, individuals with missing genotype >10%, and SNPs with minor allele frequency <0.01.

#### Data Analysis

##### Determination of Axes of Genetic Variation

Epidemiological data for this study was double entered into Microsoft^©^ Access and validated to remove duplicates. To determine whether there were significant genetic differences among the different ethnicities based on the typed SNPs that could impair association analysis, 10 (Kinnear et al., 2017) principal components (axes of genetic variation) were computed using PLINK2. A generalized linear model (GLM) was then run using the R statistical package to determine which axes of genetic variation were significantly associated with case-control status (i.e., presence or absence of TB infection). A GLM of case-control status against age, height, and weight was also run to determine their influence on association analysis.

#### Association Analysis

Association tests of SNPs with case-control status were run using PLINK1.9 with the maximum permutation test procedure used to correct for multiple testing and to determine empirical significance levels. The *--logistic* command of PLINK was used with 100,000 maximum permutations to test for significant SNPs under different models of inheritance/association (i.e., additive, dominant, recessive, heterozygote/homozygote, and genotypic), while adjusting for sex, age, weight, and height (the variable that showed significant association with case-control status by the GLM test). The association analyses were run on three separate datasets: all controls versus all cases irrespective of the infecting MTBC genotype, all controls versus cases with Maf, and all controls versus cases with Mtb TB. Association testing was run on each chromosome separately. The quality of the variants was assessed, and association analysis was run with 26 high-quality variants for the controls against all the cases put together. A total of 326 participants (145 cases; 181 controls), of which 156 were males and 170 were females, were included in the association analysis.

## Results

### Characteristics of Study Cohorts

The study population consisted of 426 individuals (191 TB individuals and 235 controls). The odds of any gender being associated with active TB in univariate logistics regression was greater for young male patients (less than 24 years; *n* = 73) and patients older than 65 years (*n* = 7) [odds ratio (OR) 12.8, 95% confidence interval (CI). 7.9–20.9, *p* = 0.001], (Table 1). Analysis of risk factors revealed that 34%, 11.6%, and 5.8% of the patients were registered as alcohol abusers, cigarette users, and substances abusers, respectively. Participants who have a pattern of drinking that interferes with their day-to-day activities were considered alcohol abusers, cigarette users were people who smoke at least once daily, and substances abusers were people who use legal and illegal drugs. Among the TB patients with available clinical data (185/191), the vast majority had pulmonary TB only (97.0%) followed by pulmonary TB with anemia (2.0%) and TB with lobar pneumonia (1.0%). At the time of sampling, 16.7% (*n* = 32) of the patients were hospitalized, and 10.9% of the patients (*n* = 21) had a previous history of TB. Analysis of risk factors revealed that 1.6% of the patients were registered diabetic TB patients. HIV-positive patients accounted for 6.8% of the study population, and 9.4% were notified as suffering from malnutrition. Sputum bacillary load at presentation was significantly higher in the Mtb group with *n* = 90 patients having sputum grades of 2+ compared with (*n* = 28) patients in the Maf group (*p* < 0.001.). Analysis of the cohort showed contributing ethnicities from the Akan, Ga-Adangbe, Ewe, and several other ethnic groups from northern Ghana.

**TABLE 1:** Demographic characteristics of patients.

Characteristics	(TB patients) N = 191	(NTB) N = 238	*p*-Value	OR	95% CI
Gender					
Male	151	54	<0.0001	12.8	7.9–20.9
Female	40	184	<0.0001	0.1	0.05–0.13
Age					
13–24	42	31	0.0195	1.9	1.1–3.2
25–34	42	41	0.2213	1.4	0.8–2.3
35–44	47	45	0.1577	1.4	0.8–2.3
45–54	40	48	0.9044	1.1	0.6–1.7
55–64	13	32	0.0270	0.5	0.2–0.9
65+	7	41	<0.0001	0.2	0.1–0.4
Risk factors					
BCG					
Yes	120	156	0.6123	0.9	0.6–1.4
No	71	82	—	—	—
Substance abuse (cigarette, alcohol, etc.)					
Yes	112	74	<0.0001	3.1	2.1–4.8
No	79	164	—	—	—
Diabetes					
Yes	3	0	0.0875	Inf	0.5-Inf
No	188	238	—	—	—
HIV					
Yes	13	0	<0.0001	Inf	3.9-Inf
No	178	238	—	—	—
Education					
Primary	33	70	0.0043	0.5	0.3–0.8
Secondary	106	112	0.0985	1.4	0.9–2.1
Tertiary	27	9	0.5610	1.4	0.5–3.4
None	25	47	0.0701	0.6	0.3–1.1
Ethnicity					
Akan	70	39	<0.0001	2.9	1.8–4.8
Ewe	28	36	0.0004	0.4	0.2–0.6
Ga	61	144	<0.0001	0.3	0.2–0.5
others	32	19	0.0065	2.3	1.2–4.5

### Genetic Structure of Pathogen Lineages and Correlation of Participants’ Demographic Variables

The 191 TB isolates after spoligotyping (S1) were identified as 78.5% (*n* = 150) Mtb and 21.5% (*n* = 41) Maf. Through stratifying by lineages (L), 86.6% (*n* = 130/150) of the Mtbss strains belonged to the Euro-American lineage (L4), with sublineages Cameroon (72.3%), Ghana (15.4%), and Haarlem (4.6%), with Uganda 11 (4.6%) being the most prevalent followed by Maf, L5 (58.5%) (*n* = 24/41). A total of 66 spoligotypes were detected; 170/191 isolates (89.0%) had previously defined shared type number (SIT). The remaining 21 isolates could not be defined by the SITVIT database and, thus, were defined as “orphan.” There was a significant correlation between case-control status and each of the demographic variables age, height, and weight-based on a generalized linear model (Table 1). None of the 10 (Kinnear et al., 2017) principal components computed showed a significant correlation with cases-control status (Table 2). On the other hand, age, height, weight, and sex showed statistically significant associations with case-control status and were, therefore, included as covariates in association tests.

**TABLE 2:** Generalized linear model (GLM) of case-control status against age, ethnicity, weight, height, sex, and principal components.

Variable	Estimate	Std error	Z-value	*p*-Value	
Age	−0.022710	0.009715	−2.338	0.0194	*
Sex	−2.043298	0.352763	−5.792	6.94 × 10^−9^	***
Ethnicity	0.031137	0.170864	0.182	0.8554	—
Weight	−0.123708	0.015236	−8.119	4.69 × 10^16^	***
Height	8.599426	1.993078	4.315	1.60 × 10^5^	***
PC1	−70.33	130.19	−0.540	0.589	—
PC2	−152.71	151.35	−1.009	0.313	—
PC3	−22.75	135.21	−0.168	0.866	—
PC4	114.29	148.67	0.769	0.442	—
PC5	71.78	123.69	0.580	0.562	—
PC6	−32.11	54.66	−0.588	0.557	—
PC7	22.90	51.79	0.442	0.658	—
PC8	68.93	103.28	0.667	0.505	—
PC9	−14.26	32.14	−0.444	0.657	—
PC10	46.72	57.52	0.812	0.417	—

Note. ***0 < *p*-value < 0.001.

*0.01 < *p*-value < 0.05.

### Genetic Association Between Tuberculosis Cases and Controls

Of the 426 samples, 10 were removed due to a questionable ethnicity status (i.e., either father or mother, or both were non-Ghanaians). Using PLINK for quality assessment of the remaining 416 samples and 29 variants (SNPs), 90 samples were removed due to missing genotype data (*--mind 0.105*), 1 variant (rs1616723) was removed due to missing genotype data (*--geno 0.105*), and 2 variants were removed due to the Hardy–Weinberg exact test—performed on control samples only (*--hwe 0.001*). No SNPs were removed as a result of minor allele frequency threshold (*--maf 0.01*). Upon alignment to the human reference genome (build 38 - GRCh38), 9 variants were changed due to allele mismatch, while the remaining 14 variants were validated for reference/alternate allele assignment. A total of 326 samples (145 cases and 181 controls; 170 females, 156 males) and 26 SNPs were retained for further analysis with a total genotyping rate of 99%.

Table 3 shows association tests results after adjustment for sex, age, height, and weight, and correction for multiple testing. All *p*-values reported are Benjamin–Hochberg adjusted (*P*_*BH*_) after 100,000 permutations. SNPs located on chromosome 4 (rs955263) in the *SORBS2* gene and on chromosome 2 (rs17235409) in *SLC11A1* encoding mannose-binding lectin were found tending to confer apparent protection (*P*_*BH*_ = 0.05) and (*P*_*BH*_ = 0.06), respectively, against TB infection irrespective of the genotype of the infecting bacteria under a recessive model of inheritance. Additionally, whereas SNP rs17048476 (on chromosome 3) tends to increase the risk of TB infection by twofold (*P*_*BH*_ = 0.07), SNPs rs8028149 (on chromosome 15) and rs5030737 decreased the risk of TB infection under a dominant model of inheritance (Table 3).

**TABLE 3:** Association tests results for controls versus all tuberculosis (TB) cases.

SNP	CHR:POS	REF/ALT	MAF	*P*_*BH*_	OR	95%CI	EMP2	MOI	Gene
rs17235409	2:218,395,009	G/A	0.06	0.13	0.135	0.13–0.15	0.06	rec	*SLC11A1*
rs17048476	3:7,987,199	G/A	0.41	0.08	2.18	2.13–2.23	0.07	dom	*LOC101927394*
**rs955263**	**4:185,697,150**	**G/A**	**0.45**	**0.05**	**0.33**	**0.32**–**0.34**	**0.05**	**rec**	***SORBS2***
rs5030737	10:52,771,482	G/A	0.04	0.11	0.25	0.24–0.26	0.09	dom	*MBL2*
rs8028149	15:61,724,906	C/T	0.57	0.08	0.43	0.42–0.45	0.08	dom	*LOC107984782*

Note. CHR:POS, chromosome and base pair position; REF/ALT, reference and alternate alleles; MAF, minor allele frequency; OR, odds ratio; CI, confidence interval; *P*_*BH*_, Benjamin–Hochberg adjusted *p*-value; EMP2, permutation test empirical *p*-value adjusted for multiple testing; MOI, mode of inheritance. The SNP in bold has the most significant association.

The bold values show the loci of interest.

### Association Analysis Between *Mycobacterium tuberculosis sensu stricto* Lineages and Controls

Similar association test procedures were performed for Mtb cases only and controls as described with 289 samples (108 cases and 181 controls) and 26 variants that passed all the aforementioned filters. Table 4 shows the association test results when considering TB caused by Mtbss. A single significant missense variant, rs17235409 in the *SLC11A1* gene, under a homozygous recessive model of inheritance appeared to confer protection against TB infection (*P*_*BH*_ = 0.04), whereas another SNP (rs8028149), under a dominant model of inheritance, was found tending to decrease the risk of Mtbss infection (*P*_*BH*_ = 0.09).

**TABLE 4:** Association tests results for controls versus *Mycobacterium tuberculosis sensu stricto* (Mtbss) cases.

SNP	CHR:POS	REF/ALT	MAF	*P*_*BH*_	OR	95%CI	EMP2	MOI	Gene
rs1524713	1:57,537,459	C/T	0.29	0.10	2.20	2.14–2.36	0.09	het	*DAB1*
**rs17235409**	**2:218,395,009**	**G/A**	**0.06**	**0.04**	**0.05**	**0.04**–**0.05**	**0.02**	**hom/rec**	***SLC11A1***
rs5030737	10:52,771,482	G/A	0.04	0.11	0.25	0.24–0.26	0.09	dom	*MBL2*
rs8028149	15:61,724,906	C/T	0.57	0.09	0.44	0.42–0.45	0.08	dom	*LOC107984782*

Note. CHR: POS, chromosome and base pair position; REF/ALT, reference and alternate alleles; MAF, minor allele frequency; OR, odds ratio; CI, confidence interval; *P*_*BH*_, Benjamin–Hochberg adjusted *p*-value; EMP2, permutation test empirical *p*-value adjusted for multiple testing; MOI, mode of inheritance. The SNP in bold has the most significant association.

The bold values show the loci of interest.

### Association Analysis Between *Mycobacterium africanum* Lineages and Controls

A single-variant rs2681052 in the gene *THSD7A*, which encodes the transmembrane protein called thrombospondin type 1 domain-containing 7A, was found tending to confer marginal protection against TB caused by Maf (EMP2 = 0.09) under a heterozygote model of inheritance.

## Discussion

Host factors are increasingly being recognized as critical for TB control considering the diversity of the outcome of interaction between MTBC and distinct human host populations. This study sought to explore potential host genetic factor (s) that may confer susceptibility or protection to distinct MTBC lineages, toward understanding crucial mechanisms of host–pathogen interaction. From this study, the most significant findings of the association analysis were two statistically significant protective SNPs: rs17235409 in *SLC11A1* against Mtb only and rs955263 in *SORBS2* against Mtb and Maf together. In addition, we found multiple marginally significant protective variants including in *SLC11A1* and *MBL2*, and younger patients <35 years and patients >65 years are associated with active TB in Ghana.

Solute carrier family 11A Member 1 (SLC11A1) (OMIM: 600266), formerly NRAMP1, a bivalent antiporter located on chromosome 2q35, delivers metal cations from the cytosol into acidic endosomal and lysosomal compartments where Fenon and Haber–Weiss reaction generates toxic antimicrobial radicals for direct antimicrobial activity against infectious microorganisms such as mycobacteria (Mulero et al., 2002; Sobota et al., 2016). SLC11A1 also appears to have multiple functions, having a role in both the resolution of infections; thus, any polymorphism in the gene may influence the function of the gene, primarily affecting the survival of the TB pathogen after phagocytosis (O'Brien et al., 2008). Of the most assessed SLC11A1 variants, the polymorphic (GT)_n_ microsatellite repeat has been shown to alter the level of SLC11A1 expression and is, therefore, a strong candidate for influencing disease incidence. Several alleles of different repeat lengths have been identified, with (GT)_n_ allele 2 conferring lower SLC11A1 expression compared with the more commonly occurring (GT)_n_ allele 3 (Zaahl et al., 2004).

Earlier studies analyzing (GT)_n_ allele 2 in humans with TB in West Africans have primarily focused on four or five polymorphisms distributed across the gene: A GT_n_ repeat in the 5′ promoter region, a four base-pair (TGTG) insertion/deletion (rs17235416) in the 3′ untranslated region (UTR), and two single-nucleotide polymorphisms (SNPs) in intron 4 (rs3731865). These mutations were found to be significantly associated with pulmonary TB. This association has been replicated in studies from Guinea-Conakry (Cervino et al., 2000) and Gambia (Yakubu et al., 2014). Bellamy et al. (Bellamy, 1998; Taype et al., 2006) found a significant association between heterozygous GC, GA, and +del genotypes, of INT4, D543N, and 3′UTR, respectively, and pulmonary TB patients, in a case-control study in Gambia. Similarly, case-control studies found an association between GA and +del genotypes, of D543N, and 3′UTR, and lung TB in Cambodia. All these genotypes were associated with resistance to TB. However, in contrast, this study identified a protective association with the more commonly occurring (GT)_n_ allele 3, exon 15 (rs17235409, D543N).

Although there are several studies on the rs17235409 variant, the role of the variant in the pathobiology of TB remains unclear. A study by Liu et al. associated the variant with treatment failure of pulmonary TB in the Chinese (Yajie et al., 2021) and Mexican populations (Salinas-Delgado et al., 2015). Among the Tibetan population in Qinghai, the variant has been associated with TB susceptibility (Xiying et al., 2016). The variant was not only associated with TB but also was implicated in other diseases as well. The rs17235409 variant was among the list of host factors associated with susceptibility to leishmaniasis (Ates et al., 2009) and rheumatoid arthritis (Niño-Moreno et al., 2017; Braliou et al., 2019). Our analysis shows that rs17235409 might be a promotor/repression gene mutation, which could lead to significant phenotypic consequences. Mutations in this gene might eventually make the phagocytic cells less toxic, thus, providing the patients with more protection from infections by Mtb. It has, therefore, been hypothesized that allele 3 would provide protection against infectious disease by driving high SLC11A1 expression and resulting in Th1-mediated immune response. It was also suspected that the intronic position of this polymorphism might affect posttranscriptional modification of the affected gene, hence, potentially affecting the resulting *SLC11A1* protein. Further analysis of the mechanism of action of *NRAMP1* and its genetic variants may lead to new approaches in controlling *tuberculosis*, which kills more people than any other disease caused by an infectious pathogen.

Sorbin and sh3 domain-containing protein 2 (*SORBS2*) gene (OMIM: 616,349) located on chromosome 4q35.1 is responsible for the regulation of many cell signaling pathways. It is involved in several biological processes such as apoptosis, mitochondrial dysfunction, and innate immunity. Our study found a protective association of rs955263 in *SORBS2* against Mtb and Maf together. Contrary to the protective value of rs955263 observed within the Ghanaian population, the variant was associated with active TB in a combined cohort from Uganda and Tanzania (36). Similar to our finding, a study from China reported an inconsistent risk association between SNPs in the *SORBS2* gene and active TB cases with respect to the data published from Uganda and Tanzania (Qi et al., 2017). The inconsistency maybe explained by different host factors that may modify the activity of the gene as well as genetic diversity observed in the different populations. There seems to be some level of population specificity, which needs to be investigated from different populations to fully understand the role of *SORBS2* gene variants in the development of active TB.

Associations between MTBC lineages and human ethnicities have been reported. Indeed, lineages 1, 2, and 4 are reported to be strongly associated with Filipino, Chinese, and “white” ethnicities, respectively (Gagneux et al., 2006; Asante-Poku et al., 2016). Likewise, in China, Hui ethnicity was found to be associated with the Beijing family of MTBC (Pang et al., 2012). Indeed, human genetic diversity has been linked to an increased or reduced susceptibility to TB. Recent studies have reported human genetic polymorphisms that influence the susceptibility to TB caused by Maf but Mtbss or vice versa. These studies indicate that human genetic susceptibility to TB is further influenced by the MTBC genotype. Conversely, a human polymorphism that reported recently on mannose-binding lectin (MBL) was associated with protection against TB caused by Maf (23). Moreover, this latter study also found Maf to bind to human recombinant MBL more efficiently, perhaps leading to an improved uptake by macrophages and selection of deficient MBL variants among human populations exposed to Maf. Although our study did not find a significant association between ethnicity and MTBC lineages, our study suggests that host genetics play an important role in TB pathogenesis, hence, the need for newer approaches to TB therapy such as host-directed immune-therapy, which have the potential to shorten the TB treatment and prevent resistance by promoting autophagy.

## Conclusion

In conclusion, we found SLC11A1 as a potential susceptibility gene of substantial interest for TB caused by *M. africanum*, which is an important pathogen in West Africa. Given the odds ratio specified in the sample size calculation method (which effectively powers the study to detect loci that increase TB susceptibility), it implies that further studies on larger case-control sample sizes are needed to confirm the resistance loci observed in this study.

## Data Availability

The datasets presented in this article are not readily available because data cannot be shared publicly because of patient confidentiality. Data are available from the Noguchi Memorial Institutional Data Access/Ethics Committee (contact *via* GWemakor@noguchi.ug.edu.gh) for researchers who meet the criteria for access to confidential data. Requests to access the datasets should be directed to GWemakor@noguchi.ug.edu.gh.

## Supplementary Material

Supplementary Table S1 Descriptive statistics of the allelic frequencies of SNPs in different human populations.
