# Supplementary material for: Genetic Analysis of TB Susceptibility Variants in Ghana Reveals Candidate Protective Loci in SORBS2 and SCL11A1 Genes
Source: Front Genet. 2022 Feb 15;12:729737. doi: 10.3389/fgene.2021.729737 (PMC8886735; doi:10.3389/fgene.2021.729737)
Supplement: Supplementary file 1 [file Table1.pdf]

Supplementary Table 1: Descriptive statistics of the allelic frequencies of SNPs in different human populations

			Current study		Allelic frequencies of SNPs in different human populations from the 1000 Genomes Project					
Gene	rs number	Allele	counts	frequency	All	AFR	AMR	EAS	EUR	YRI
CALN1	rs844669	A	593	0,74	0,68	0,72	0,57	0,75	0,61	0,74
		C	206	0,26	0,32	0,28	0,43	0,25	0,39	0,26
SLC11A1	rs17235409	A	146	0,18	0,07	0,06	0,08	0,14	0,01	0,06
		G	653	0,82	0,93	0,94	0,92	0,86	0,99	0,94
LOC107984706	rs857063	A	600	0,75	0,66	0,67	0,57	0,47	0,82	0,66
		C	199	0,25	0,34	0,33	0,43	0,53	0,18	0,34
F13A1	rs1482868	C	664	0,83	0,69	0,75	0,74	0,76	0,66	0,75
		T	135	0,17	0,31	0,25	0,26	0,24	0,34	0,25
PLCD4	rs3731869	G	553	0,69	0,08	0,12	0,02	0,12	0,03	0,14
		T	246	0,31	0,92	0,88	0,98	0,88	0,97	0,86
MBL2	rs5030737	A	44	0,05	0,03	0	0,03	0	0,06	0
		G	755	0,95	0,97	100	0,97	100	0,94	100
ZPLD1b	rs12636260	G	295	0,73	0,54	0,73	0,53	0,54	0,45	0,76
		T	107	0,27	0,46	0,27	0,47	0,46	0,55	0,24
F13A1	rs1482868	C	277	0,70	0,69	0,75	0,74	0,76	0,66	0,52
		T	120	0,30	0,31	0,25	0,26	0,24	0,34	0,48
DAB1	rs1524713	C	295	0,72	0,69	0,75	0,74	0,76	0,66	0,75
		T	116	0,28	0,31	0,25	0,26	0,24	0,34	0,25
Bsm-I	rs1544410	C	313	0,74	0,7	0,73	0,74	0,94	0,6	0,7
		T	108	0,26	0,3	0,27	0,26	0,06	0,4	0,3
GLO1	rs1616723	C	55	0,17	0,12	0,18	0,05	0,18	0,08	0,19
		T	271	0,83	0,88	0,8	0,95	0,8	0,9	0,81

						2		2	2	
AK124857	rs17048476	A	165	0,40	0,24	0,4	0,18	0,08	0,21	0,37
		G	245	0,60	0,76	0,6	0,82	0,92	0,79	0,63
SLC11A1	rs17235409	A	23	0,05	0,07	0,06	0,08	0,14	0,01	0,06
		G	408	0,95	0,93	0,94	0,92	0,86	0,99	0,94
RBFOX1	rs2346943	G	89	0,21	0,33	0,3	0,38	0,26	0,42	0,29
		T	331	0,79	0,67	0,7	0,62	0,74	0,58	0,71
ADAMTS14	rs2587469	A	226	0,58	0,62	0,56	0,5	0,7	0,62	0,6
		G	165	0,42	0,38	0,44	0,5	0,3	0,38	0,4
THSD7A	rs2681052	A	155	0,38	0,5	0,38	0,64	0,41	0,64	0,39
		C	248	0,62	0,5	0,62	0,36	0,59	0,36	0,61
SLC11A1	rs2695342	G	422	0,97	0,98	0,94	100	100	100	0,96
		A	12	0,03	0,02	0,06	0	0	0	0,04
SLC11A1	rs2807348	A	330	0,78	0,45	0,72	0,41	0,33	0,26	0,77
		G	92	0,22	0,55	0,28	0,59	0,67	0,74	0,23
RGS3	rs3860173	C	234	0,57	0,63	0,65	0,62	0,39	0,75	0,59
		T	177	0,43	0,37	0,35	0,38	0,61	0,25	0,41
SFRP1	rs4236914	C	283	0,72	0,79	0,73	0,79	0,72	0,95	0,68
		T	111	0,28	0,21	0,27	0,21	0,28	0,05	0,32
SLC38A4	rs4768760	A	243	0,58	0,61	0,59	0,61	0,61	0,66	0,59
		C	174	0,42	0,39	0,41	0,39	0,39	0,34	0,41
ADGRL3	rs4860106	A	102	0,40	0,49	0,63	0,61	0,21	0,59	0,63
		G	156	0,60	0,51	0,37	0,39	0,79	0,41	0,37
UBLCPI, IL12B	rs4921437	C	283	0,68	0,86	0,74	0,89	100	0,83	0,76
		T	130	0,32	0,14	0,26	0,11	0	0,17	0,24
WNT4	rs557438	A	117	0,30	0,23	0,33	0,12	0,15	0,23	0,29
		G	279	0,70	0,77	0,6	0,88	0,8	0,7	0,71

						7		5	7	
GRIN2B	rs7297313	A	250	0,64	0,78	0,64	0,87	0,8	0,86	0,61
		C	143	0,36	0,22	0,36	0,13	0,2	0,14	0,39
Taq-1	rs731236	A	306	0,76	0,72	0,71	0,74	0,93	0,6	0,69
		G	99	0,24	0,28	0,29	0,26	0,07	0,4	0,31
VPS13C	rs8028149	C	166	0,43	0,65	0,44	0,77	0,84	0,67	0,44
		T	224	0,57	0,35	0,56	0,23	0,16	0,33	0,56
SH3TC2	rs930205	C	170	0,41	0,51	0,37	0,49	0,65	0,58	0,39
		T	242	0,59	0,49	0,63	0,51	0,35	0,42	0,61
SORBS2	rs955263	A	187	0,45	0,42	0,43	0,47	0,5	0,23	0,41
		G	226	0,55	0,58	0,57	0,53	0,5	0,77	0,59
ABCA8	rs9893385	A	231	0,56	0,52	0,53	0,57	0,51	0,41	0,58
		G	183	0,44	0,48	0,47	0,43	0,49	0,59	0,42

AFR: Africa, AMR: America, EAS: East Asia, EUR: Europe, YRI: Yoruba
